# Self-administered gerocognitive examination (SAGE) aids early detection of cognitive impairment at primary care provider visits

**DOI:** 10.3389/fmed.2024.1353104

**Published:** 2024-06-13

**Authors:** Douglas W. Scharre, Nicole E. Vrettos, Haikady N. Nagaraja, Randell K. Wexler, Aaron D. Clark, Christopher M. Nguyen

**Affiliations:** ^1^Division of Cognitive Neurology, Department of Neurology, The Ohio State University Wexner Medical Center, Columbus, OH, United States; ^2^Division of Biostatistics, College of Public Health, The Ohio State University, Columbus, OH, United States; ^3^Department of Family and Community Medicine, The Ohio State University Wexner Medical Center, Columbus, OH, United States; ^4^Department of Psychiatry and Behavioral Health, The Ohio State University Wexner Medical Center, Columbus, OH, United States

**Keywords:** mild cognitive impairment, geriatric assessment, self-administered cognitive screening, primary care provider, SAGE, early detection

## Abstract

**Introduction:**

Current estimates indicate that up to 50–75% of dementia cases are undiagnosed at an early stage when treatments are most effective. Conducting robust accurate cognitive assessments can be time-consuming for providers and difficult to incorporate into a time-limited Primary Care Provider (PCP) visit. We wanted to compare PCP visits with and without using the self-administered SAGE to determine differences in identification rates of new cognitive disorders.

**Methods:**

Three hundred patients aged 65–89 without diagnosed cognitive disorders completing a non-acute office visit were enrolled (ClinicalTrials.gov identifier: NCT04063371). Two PCP offices conducted routine visits for 100 consecutive eligible patients each. One office used the SAGE in an additional 100 subjects and asked available informants about cognitive changes over the previous year. Chart reviews were conducted 60 days later. One-way analysis of variance and Fisher exact tests were used to compare the groups and outcomes.

**Results:**

When SAGE was utilized, the PCP documented the detection of new cognitive conditions/concerns six times (9% versus 1.5%) as often (*p* = 0.003). The detection rate was nearly 4-fold for those with cognitively impaired SAGE scores (*p* = 0.034). Patients having impaired SAGE score and informant concerns were 15-fold as likely to have new cognitive conditions/concerns documented (*p* = 0.0007). Among providers using SAGE, 86% would recommend SAGE to colleagues.

**Discussion:**

SAGE was easily incorporated into PCP visits and significantly increased identification of new cognitive conditions/concerns leading to new diagnoses, treatment, or management changes. The detection rate increased 15-fold for those with impaired SAGE scores combined with informant reports.

## Introduction

The number of Americans aged over 64 is projected to nearly double from 52 to 95 million between 2018 and 2060, and the over 64 age group’s share of the total population will rise from 16 percent to 23 percent (1). It has been estimated that the prevalence of dementia and mild cognitive impairment (MCI) among individuals 65 years and older in the US in 2016 was 10 and 22%, respectively (2). While there is a wide range of estimates of the prevalence of MCI among older adults ages 65 and above and it can be as high as 29.3% among those 85 years and older (3, 4), early detection of cognitive impairment is beneficial to discover potentially reversible conditions (5), initiate treatments, and allow individuals to play an active role in decision-making planning for their future (6).

Primary care providers (PCPs) are typically the first to identify and evaluate patients with neurocognitive disorders such as MCI and dementia. However, the diagnosis of cognitive impairment has historically been challenging to establish in primary care settings (7–9). Conducting robust accurate cognitive assessments can be time-consuming for providers and difficult to incorporate into a time-limited visit. Additionally, while there are numerous validated cognitive screening assessments, most require an experienced and trained administrator with time to conduct the assessment. These personnel resources are limited in many primary care offices. In a recent survey, 50% of PCPs reported that they, or their staff, administered a standardized cognitive screening to about half of their patients with cognitive concerns (10). Barriers to performing cognitive testing identified by PCPs in the survey included lack of expertise to assess cognitive measures, cost, and time (10). Another barrier is the limited reimbursement by Medicare for brief cognitive screening evaluations (11). In addition, patients may not report and PCPs may not notice the subtle cognitive deficits of MCI in routine patient visits without objective cognitive assessments. About 50–75% of dementia cases are undiagnosed at an early stage when treatmens are most effective and take, on average, 32 months to be identified after symptom onset (12). Identifying cognitive impairment is often delayed so long that the recent FTA-approved disease-modifying therapies for MCI due to Alzheimer’s disease (AD) and for mild AD dementia are less effective and may be outside the window of approved use.

Ideally, a cognitive screening test or case finding tool for use in a primary care practice should be brief, inexpensive, easily administered and scored, and exhibit high sensitivity and specificity for identifying cognitive changes (13, 14). An objective cognitive assessment or obtaining information about cognition from the patient or informant is required as part of an Annual Wellness Visit as a benefit for Medicare recipients (15). While there are numerous cognitive screening tests with good psychometric properties to differentiate between dementia and healthy controls (16–18), they are often underutilized due to the demand for clinical resources for administration (19). Most have not been evaluated for efficacy in detecting MCI from normal aging (20). Informant-based measures have also been utilized in these settings, although can be limited due to the lack of accessible family members or acquaintances (21, 22). More abbreviated screening measures have also been introduced in primary care settings to be followed by more sensitive tests (23).

To address some of the barriers to cognitive testing in primary care settings, the Self-Administered Gerocognitive Examination (SAGE) was developed to detect early signs of cognitive impairment (24). SAGE assesses multiple cognitive domains (orientation, language, calculations, memory, abstraction, executive, and visuospatial) with scores ranging from 0 to 22. It has four equivalent interchangeable alternate versions, age and education norms and allows for completely unsupervised self-administration without involvement of a technician or clinician which minimizes staff involvement and makes it very efficient to use in PCP settings. It is completed in an average of 13 min, takes 20 s for scoring, and can be given in any setting (24, 25). SAGE has been validated for MCI with an Area Under the ROC curve of 0.92 to accurately detect cognitively impaired subjects (95% specificity and 79% sensitivity) (24) and therefore can identify individuals who may be appropriate for the new disease-modifying AD therapies. It has been compared with other commonly used office-based multidomain brief cognitive tests (14). It can predict MCI conversion to dementia at least 6 months sooner than the Mini-Mental State Examination (MMSE) (25). Therefore, while SAGE may not pick up the earliest cogntive deficits as well as standard multi-hour neuropsychological batteries, it does better than less robust brief cogntive tests that have fewer domains assessed. SAGE (available free of charge at: sagetest.osu.edu) has been converted to digital format (i.e., eSAGE or BrainTest^®^; available at: https://braintest.com) with all the features of self-administration and equivalency (14). The eSAGE (BrainTest^®^) is automatically scored for the provider with established standardized and automatic age and education normalized core-lab central scoring, and HIPAA compliance. SAGE or eSAGE can be used to establish a baseline score to detect changes when given longitudinally over time.

The purpose of this study was to evaluate the utility of SAGE in primary care settings for detecting early signs of cognitive impairment among older adults. Given the projected increase in the aging population and the high prevalence of MCI and dementia, early detection is crucial for effective treatment and planning. The study aims were to determine whether the implementation of SAGE can improve the identification of cognitive issues during routine primary care visits, compared to standard practices that do not use SAGE. The study also assessed the practicality and ease of use of SAGE for primary care providers (PCPs) and their staff.

## Materials and methods

### Study cohort

Individuals aged 65–89 who arrived for a non-acute care office visit and had no prior evidence of an MCI or dementia diagnosis in their medical records were enrolled in this study. This investigational study met institutional requirements for conduct of human subjects research, was approved by The Ohio State University’s Institutional Review Board, and was pre-registered on ClinicalTrials.gov (identifier: NCT04063371). Participants were recruited from two primary care offices associated with The Ohio State University Wexner Medical Center. One location served as the intervention office where all providers used a standardized method for screening for cognitive impairment. This consisted of using SAGE and having an informant interview (when possible) to ascertain if a significant change (based on the primary care provider’s opinion) occurred in the patient’s cognitive abilities over the previous year. While the intervention office could have used SAGE or eSAGE (BrainTest^®^), they chose to use the paper form of SAGE, primarily due to familiarity with the paper SAGE. The second location served as the control office where all providers continued to conduct their visits, including any screening for cognitive impairment, based on their standard practices (which did not include the use of SAGE or eSAGE). Study data were collected and managed using REDCap electronic data capture tools hosted at The Ohio State University (26, 27).

The study consisted of three groups: the intervention group, control group 1, and control group 2. The intervention group consisted of patients seen by the intervention office who completed the SAGE during their appointment. Control group 1 consisted of patients seen by providers in the control office where SAGE was not utilized. Control group 2 consisted of patients seen by the intervention office who did not complete SAGE during their appointment due to time constraints, patient noncompliance, and provider oversight.

### Study design

Chart reviews were conducted on patients with scheduled PCP appointments from October 2019–January 2020. The charts were reviewed before the patient visits to assess eligibility and in sequential order based on the appointment date/time until 100 eligible patients were enrolled in each group. None of the control group patients had any documentation of past completion of SAGE or eSAGE in their medical records. Two patients in the intervention group had prior SAGE use documented in their medical records. None of the intervention group patients had prior eSAGE use documented in their medical records.

For participants who met the inclusion/exclusion criteria, the charts were reviewed by two reviewers 60+ days later using a 60-day window from the initial visit. The reviewers conducted the chart reviews via a standardized method to allow for consistency. The provider’s notes were reviewed to determine if there was a documented concern for cognitive impairment. If a concern for cognitive impairment was identified, the charts were reviewed further to assess the number of referrals for further evaluation/management of potential cognitive impairment (e.g., lab work, neuroimaging, neuropsychology testing, neurology/psychiatry consultations, occupational, physical, and speech therapies, counseling, respite care, day care, home health, social work, legal and financial planning, and cognitive research), the initiation of pharmacological interventions, and the diagnosis of a cognitive disorder. Charts were also screened to assess any follow-up visits scheduled regarding cognitive issues. Results from SAGE and the PCP’s judgment of the informant information regarding the patient’s cognitive change over the previous year were obtained for the intervention group. A trained research assistant scored all completed SAGEs. The providers in the intervention office were also asked to score the SAGE on their own for each of their patients.

The researchers did not provide specific instructions or guidance to the providers in the intervention office on how to incorporate the SAGE into their visits. The process therefore varied for each provider allowing for real world generalizability. The providers were queried at the end of the trial on the processes they used. All providers had the SAGE scoring instructions and copies of the SAGE at their workstation. Each provider worked with their own medical assistants to develop a strategy that worked best for them. The SAGE was always passed out by the provider or medical assistant and was never distributed by the check-in staff. All SAGEs were completed in the office, usually in an exam room. The timing of SAGE administration varied depending on the workflow that day. If a provider was running late, it was often completed before the visit and if a provider was on time it was often completed at the end of the visit.

Upon completion of the trial, the providers from the intervention group were requested to complete a questionnaire (Supplementary Figure S1) to evaluate the practicality and ease of use of SAGE.

The following outcome measures were collected: (1) the proportions of patients in the intervention and the control group with an identified concern for new cognitive impairment or a diagnosis of a new cognitive disorder; (2) differences in SAGE scoring between the intervention office and trained research assistant; (3) and results of the questionnaire to evaluate the practicality and ease of use of SAGE by PCPs and staff.

### Sample size choice

The study was powered to compare proportions of two groups (control vs. SAGE intervention). With anticipated proportions of diagnosis of 0.05 (=1/20) for the control and 0.17 (28) for the SAGE intervention group, and chosen sample sizes of 100 each, a two-sample test of proportions carried out with a two-tailed level of significance of 0.05 will have 78% power to detect the difference. Comparison test between the combined control (*n* = 200) and SAGE (*n* = 100) will have 89% power.

### Statistical analyses

A one-way analysis of variance and Fisher exact tests were used to compare the groups and outcomes. Relative risk (RR) estimates and associated 95% confidence intervals (CIs) were computed. The level of significance was set at 0.05. The JMP Version 17 (SAS Institute, Cary NC) was used for statistical analyses.

## Results

### Demographics

A total of 300 patients were enrolled across all three groups: intervention group (*n* = 100), control group 1 (*n* = 100), and control group 2 (*n* = 100). Demographic characteristics and clinical characteristics are presented in Table 1. There were no significant differences in age, race, or sex between the intervention group (*n* = 100) and the combined control subgroups (*n* = 200).

**Table 1:** Demographic and clinical characteristics.

Characteristic	Group			*p*-value for comparison^	
	Intervention (I) (*n* = 100)	Control 1 (C1) (*n* = 100)	Control 2 (C2) (*n* = 100)	I, C1, C2 (3 groups)	I, C1 + C2 (2 groups)
Age, mean yrs. (SD) Range	72.47 (5.64) 65–87	72.12 (6.25) 65–90	72.66 (5.98) 65–89	0.8193*	0.9105*
Sex, % female	58%	57%	67%	0.2892^#^	0.5321^#^
Ethnicity				0.0276^#^	0.1409^#^
White	83%	82%	74%		
Black	16%	10%	22%		
Other	1%	8%	4%		
Hypertension (%yes)	71%	73%	81%	0.2222^#^	0.2616^#^
Hyperlipidemia (%yes)	80%	73%	88%	0.0278^#^	1.0000^#^
Diabetes (%yes)	25%	31%	45%	0.0100^#^	0.0277^#^
Heart disease (%yes)	48%	39%	55%	0.0832^#^	0.9027^#^
Obesity (%yes)	52%	51%	63%	0.1656^#^	0.4603^#^
At least 2 comorbidities above (%yes)	85%	77%	90%	0.0445^#^	0.8676^#^
At least 3 comorbidities above (%yes)	61%	60%	75%	0.0465^#^	0.3035^#^
Number of medications mean (SD) and range	9.31(5.36) 0–29	9.86(6.47) 0–28	12.50(6.13) 3–34	0.0004*	0.0083*
SAGE score mean (SD) and range	17.8 (3.8) 2–22	Not measured			

^ Three-group comparison is for all three arms and two-group is for the intervention versus combined control arms; SD, Standard deviation; ^*^ Welch test; ^#^ Fisher exact test.

Among common chronic conditions often co-occurring with dementia (29) there were no significant differences in proportions in those having hypertension, hyperlipidemia, heart disease, or obesity between the intervention group and the combined control subgroups. We did find significantly less diagnosis of diabetes and mean total number of medications in the intervention group compared to the combined control subgroups.

### Findings from SAGE

**Figure 1:**
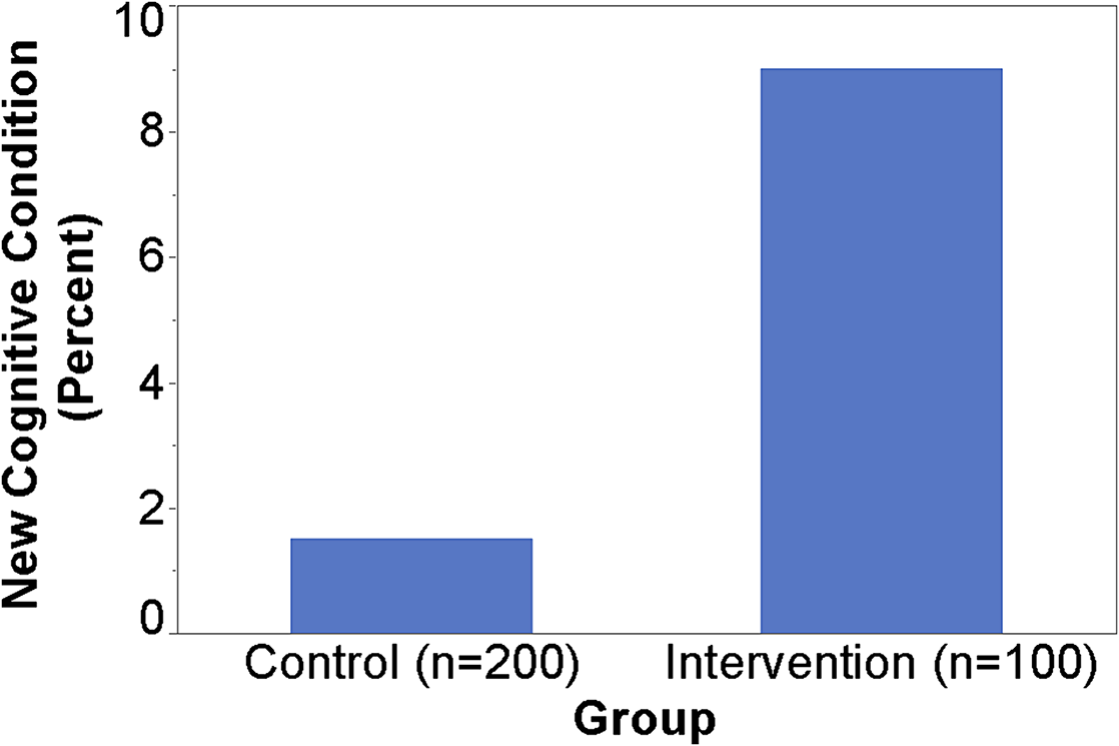
Detection of new cognitive conditions/concerns by providers.

**Figure 2:**
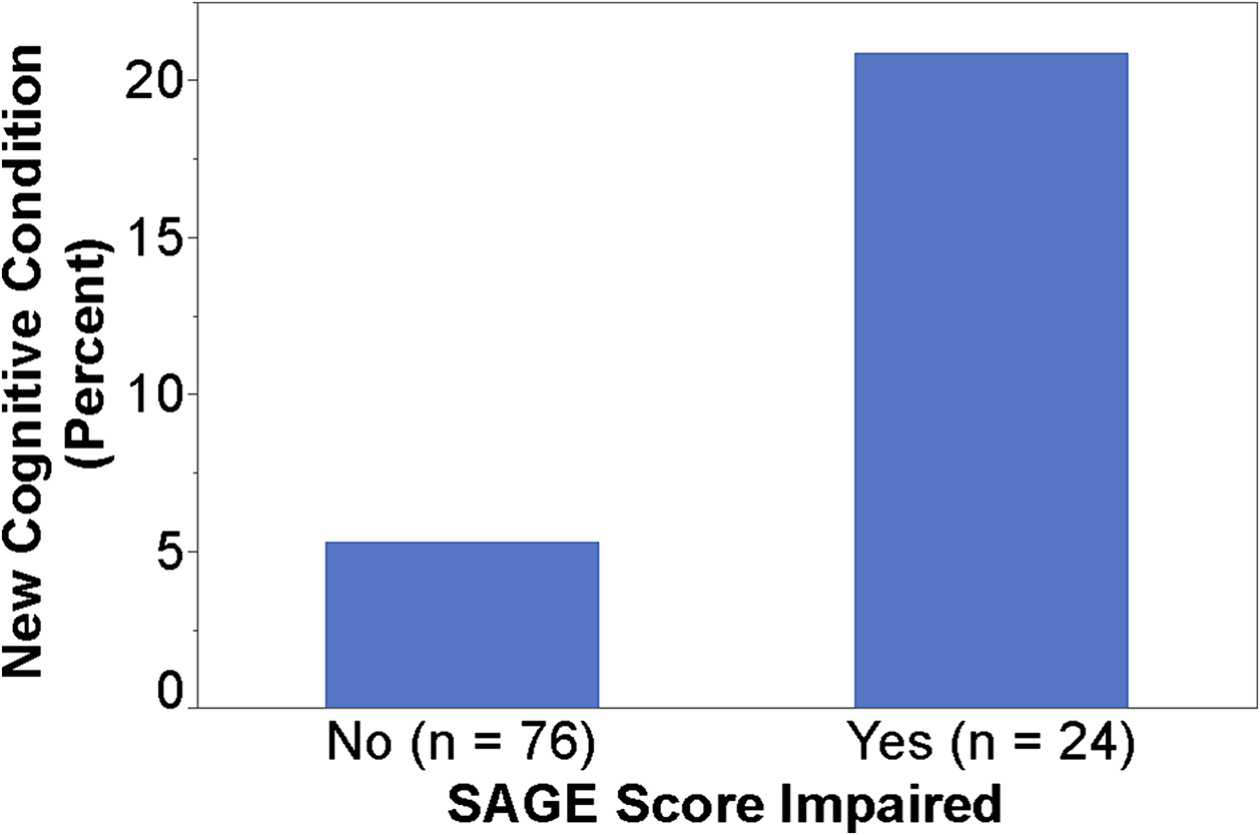
Detection rate of new cognitive conditions/concerns in the Intervention group by normal or impaired SAGE scores.

**Figure 3:**
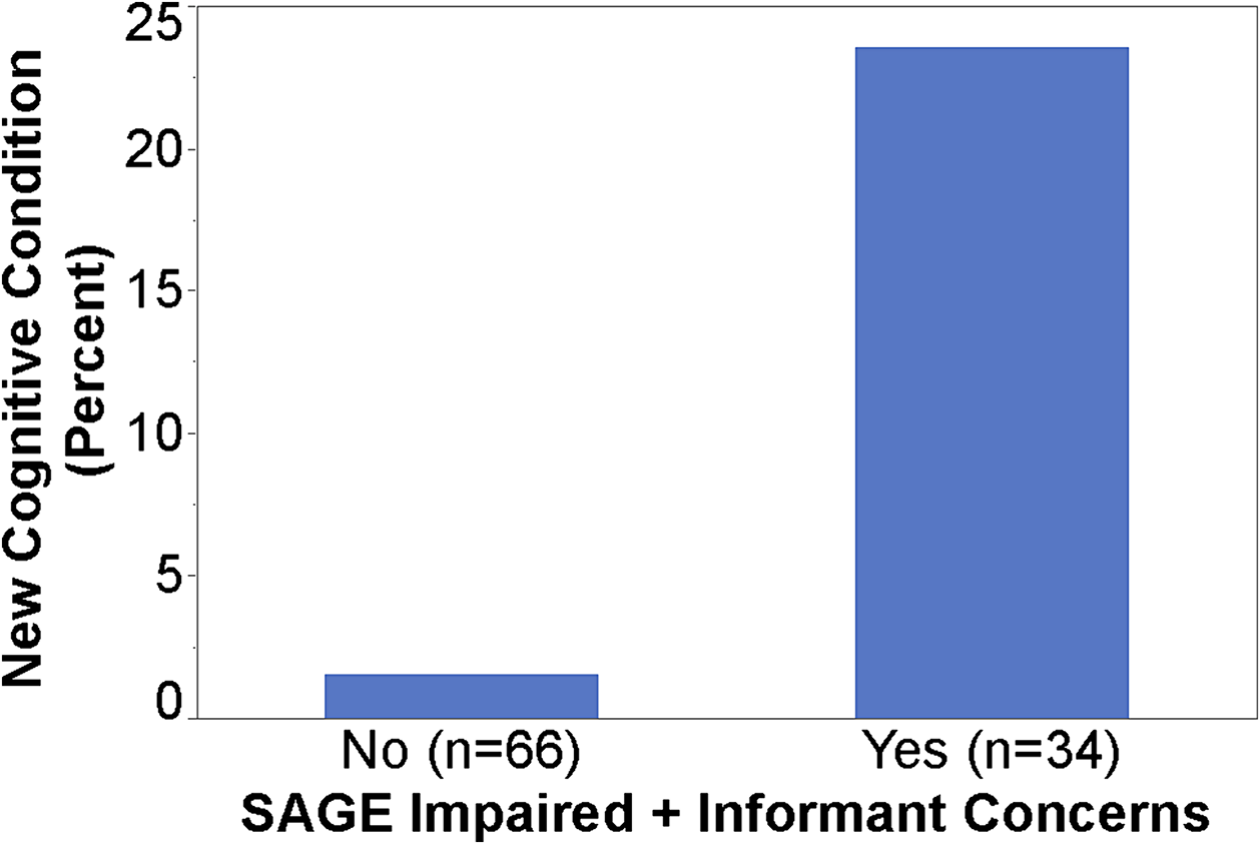
Detection rate of new cognitive conditions/concerns in the Intervention group by impaired SAGE scores/informant concerns or not.

When SAGE was utilized, the providers documented the detection of new cognitive conditions/concerns six times (9% versus 1.5%) as often (*p* = 0.003); 95% CI for the RR was (1.66, 21.68) (Figure 1). The intervention group detection rate of new cognitive conditions/concerns was 3.96-fold for those with cognitively impaired SAGE scores (researcher graded SAGE score < 17) when compared to those with normal SAGE scores (researcher graded SAGE score of 17 or higher); 5 out of 24 versus 4 out of 76 (*p* = 0.034), and 95% CI for the RR was (1.15, 13.57) (Figure 2). The 34 individuals having either impaired SAGE scores or informant concerns of a significant change in the patient’s cognitive functioning over the previous year were 15.5-fold as likely to have new cognitive conditions/concerns documented; 8 out of 34 versus 1 out of 66 (*p* = 0.0007), and 95% CI for the RR was (2.02, 119.10) (Figure 3).

Of the 53 out of the 100 patients in the intervention group with informant information documented, 17 had cognitively impaired SAGE scores and 36 had normal SAGE scores. In 35% of those with cognitively impaired SAGE scores and in 28% of those with normal SAGE scores, the informants had expressed concerns about significant cognitive change over the last year. While the former proportion is higher, there is no significant difference between these two proportions (*p* = 0.750). Overall, 16 out of 53 informants (30%) expressed concerns about significant cognitive change over the last year.

Charts were reviewed for a 60-day window after the initial patient visit. During that time, 4% of patients in the intervention group, 1% in control group 1, and none in control group 2 had at least one PCP follow-up appointment scheduled for additional investigation of a cognitive impairment disorder (*p* = 0.132). When the two control groups were combined, the difference between the intervention (4%) and the combined control group (0.5%) became statistically significant (*p* = 0.044). Seven percent of patients in the intervention group, 2% in control group 1, and none in control group 2 had at least one referral for further evaluation/management of potential cognitive impairment (*p* = 0.012). Further, the difference between the intervention (7%) and the combined control group (1%) was highly significant (*p* = 0.007). Five percent of patients in the intervention group, 1% in control group 1, and none in control group 2 had more than one referral ordered. Referrals were sent for neuroimaging, laboratory evaluations, neurology/psychiatry consultation, legal assistance, home health, social work, financial planning, and counseling. In addition, in some cases, the chart reviews revealed cognitive medication suggestions, home health discussions, discussions to consider stopping driving, and documentation of previous laboratory and neuroimaging results. No patients in any group were started on a pharmacological intervention to manage cognitive impairment.

### Findings from provider questionnaire

Fourteen providers saw patients in the intervention office, and ten providers saw patients in the control office. Of the 14 providers in the intervention office, 11 utilized SAGE and were given the opportunity to complete the questionnaire (Supplementary Figure S1). Ten of those providers were physicians (5 female) and one was a Certified Nurse Practitioner (female) with four having 1–9 years, four having 10–19 years, and three having over 20 years of practice experience. Out of the eight providers who finished the questionnaire, 25% felt it took up too much time during routine visits, 75% thought that it was useful, 63% felt it influenced their decision to further evaluate for cognitive impairment, and 63% thought that it led to more confidence regarding the presence or absence of cognitive impairment. Overall, 86% (6 out of 7) of providers indicated that they would recommend SAGE to be used during office visits, and that it can be better implemented if given outside exam rooms and at Annual Wellness Visits to prevent workflow disruption and allow for discussion time.

### SAGE scoring discrepancies

**Figure 4:**
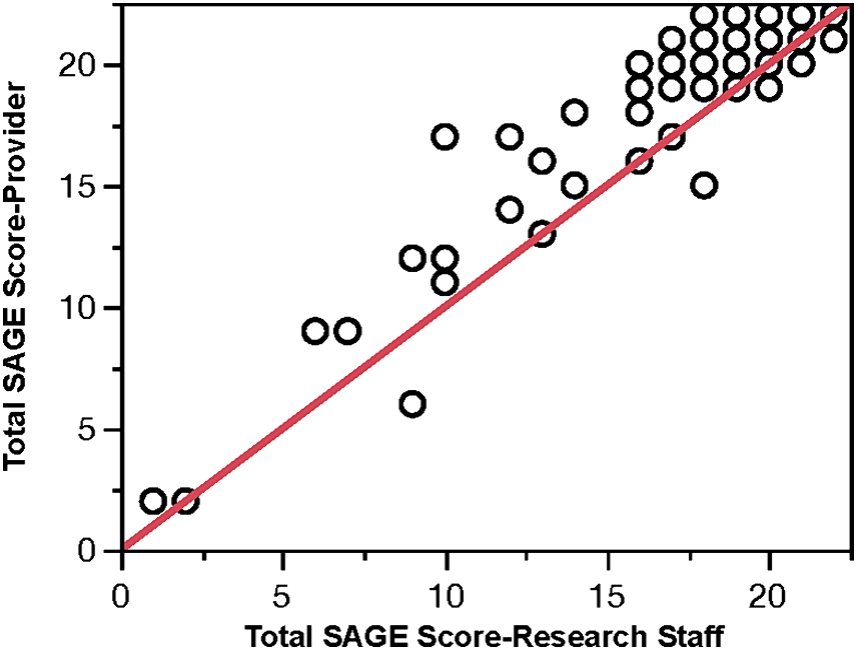
PCP SAGE score vs. research staff score (red line is the 45° line).

Eighty-three SAGEs were scored by the providers in the intervention office. The providers reviewed the other 17 SAGEs performed, but they did not have a score documented. The scoring discrepancies (the researcher scored SAGE minus the provider scored SAGE) ranged from −7 points to +3 points. Sixty-six percent of the total score discrepancies were within ±2 points. The mean difference of −1.45 was significantly different from 0 (*p* < 0.0001), with a 95% CI (−1.10, −1.79). Overall, the providers scored the SAGE incorrectly 78% of the time. Figure 4 plots the PCP SAGE scores against the researcher’s score. It suggests that providers’ scores are likely to be higher. The Spearman correlation between these scores was 0.843. The most significant discrepancies between the provider and researcher subscores occurred with the problem-solving question (40%) and the 3-D figure question (29%).

## Discussion

Disease-modifying therapies for MCI due to Alzheimer’s disease and for mild AD dementia are now FDA-approved. However, identifying cognitive impairment is often delayed so long that these treatments are less effective and may be outside the window of approved use. Expert panels have continued to stress the need for validated, brief, case-finding cognitive assessment tools, especially self-administered tests that allow for unsupervised administration and accurately identify those with MCI (30). It is critical to focus on those individuals still in the MCI stage because a treatment that prevents MCI due to AD or other neurodegenerative diseases from progressing to dementia would significantly impact quality of life, caregiver burden, and cost of care. Diagnosing AD during the MCI stage could save the nation as much as $7 trillion in medical and long-term care expenditures for those alive in 2018 that will develop AD (31). Having easy access to low/no-cost validated cognitive assessments that can be taken completely unsupervised in any setting will allow for earlier identification of cognitive impairment.

Outside of delirium, cognitive impairment often starts insidiously and progresses slowly. These slowly progressive cognitive disorders are challenging to be identified at an early stage due to the insidious nature of their progression. Besides the immediate family members, the PCP is next in a position to be able to identify new cognitive impairments. This identification is often challenging for the PCP as the patient may have decreased insight and not bring up their cognitive changes, the patient may not come with an informant or family member, the office may not have enough dementia-knowledgeable personnel resources required to administer testing, and the time needed for cognitive assessments are time-consuming and difficult to incorporate into time-limited PCP visits. For all these reasons, individuals with cognitive impairment are often identified too late for early treatments to be most successful. These issues, among others, are responsible for why 50–75% of dementia cases are undiagnosed, untreated, and unmanaged (12).

This study found that SAGE can be easily incorporated into PCP visits. It significantly increased awareness of new cognitive conditions/concerns that could lead to new diagnoses, treatment, or management changes. If by using SAGE, an MCI or dementia condition is identified by the PCP, there is then clear direction to consider evaluations for treatable causes of cogntive impairment. Typically blood tests to include TSH and B12 and a neuroimage (CT or MRI) might be ordered (5). If these are all negative and a neurodegenerative disorder is considered, further consultation with specialists may be indicated.

PCP use of SAGE resulted in a 6-fold detection of new cognitive conditions/concerns. This suggests that SAGE at least “started the conversation” between the patient and the PCP regarding their cognitive concerns or issues. Approximately 63% of the PCPs felt SAGE influenced their decision to further evaluate for cognitive impairment. This was borne out in the data where we found those that scored in the cognitively impaired range on SAGE (score < 17) were four times as likely to be reported by the PCP to have a new cognitive issue than those scoring in the normal range. This suggests that low SAGE scores can be an important triggering factor for the PCP to identify MCI or early dementia that may have been missed in a routine PCP visit that did not use SAGE.

Our patient population (Table 1) did not show significant differences in demographic or other clinical characteristics between the intervention and the combined control groups except for the findings of significantly less diagnosis of diabetes and total number of medications in the intervention group. So, there is no evidence that the high detection of cognitive impairment in patients undergoing SAGE was due to a high prevalence of chronic diseases or polypharmacy which may affect their cognitive performance.

The importance of having an informant that knows the patient well cannot be understated. In this study, in the intervention group, an informant accompanied the patient to the PCP visit 53% of the time. The informant was asked if they felt a significant change had occurred in the patient’s cognitive skills over the previous year. If the PCPs opinion was that the informant was describing a significant change, then a new cognitive condition/concern was more likely recorded by the PCP. In fact, those patients who either were noted to have a SAGE score in the cognitively impaired range (<17) or whose informant persuaded the PCP to agree that the patient had a significant cognitive change in the last year, were 15-fold as likely to be identified as having a new cognitive issue by the PCP.

PCPs are committed to help identify cogntive deficits, diagnose, treat, and manage their patients. However, they often have limited resources and time. SAGE or eSAGE have several qualities that allow it to be used efficiently in PCP office visits. This study indicated that SAGE can be administered with little disruption to the office workflow of PCPs. When there are time constraints, it was suggested that the individual finish up the test in a private area outside an exam room and turn SAGE in before they leave. Just like a laboratory test or scan, the results could be relayed to the patient in a couple of days, and if indicated, another appointment could be set up to review the results, giving more time to address their patient’s cognitive issues. The PCPs also suggested that SAGE may work best at Annual Wellness Visits, where they have more time allotted to the patient to be able to give the test. Because SAGE or eSAGE is given unsupervised, with the instruction simply to “do the best you can,” it eliminates the need to train knowledgeable staff to administer the test. Given all the barriers to performing cognitive assessments in the PCP office visit setting, the fact that 86% of the PCPs in this study indicated that they would recommend SAGE to colleagues bodes well for SAGE being accepted and used more routinely in PCP offices.

Finally, in this study, we also investigated the ability of the PCP to accurately score SAGE when given detailed written scoring instructions (available on the website at: https://sagetest.osu.edu) and a one-time in-person discussion of how to score SAGE. Although many times the score from the PCP was close to the actual score, we found that they provided an incorrect score (typically higher) 78% of the time, which downplays cognitive deficits. The mean difference was −1.45 points, and 23% of the time, the score was off by three or more points. A difference of 2 or more points for the SAGE is clinically significant (25).

Even if a cognitive test is valid and accurate in detecting cognitive impairment in the research setting, it is often a different situation when used in a PCP office where staff is often not as trained or as knowledgeable as they should be regarding cognitive assessment tools. This leads to errors in the administration of a test and errors in the scoring of the assessment. The self-administered nature of SAGE means that staff can avoid any mistakes in administration. However, this study points out the error rates in PCP scoring, despite the relatively straightforward scoring instructions. For SAGE, questions are scored either correct or incorrect (1 or 0 scores), or they are scored as fully correct, partially correct, or incorrect (2 or 1 or 0 scores). Accuracy is diminished when scoring mistakes are made, impacting the interpretation of the test results. When an individual takes SAGE repeatedly over time, scoring inaccuracies also impact perceived changes over time. While emphasizing proper scoring to PCPs may reduce errors, another solution is for PCPs to utilize the digital format of SAGE, eSAGE (also known as BrainTest^®^, available at: https://braintest.com). It can send HIPPA-compliant automatically scored results to the PCP office (14). Being self-administered and utilizing centralized scoring will reduce errors and increase the accuracy of the test.

There are several limitations of our study. We do not have any longitudinal follow up of our subjects and do not have any further knowledge of their ultimate cognitive diagnoses. Every PCP office will have different patient clinic processes and office environments that may influence and create a different experience in using SAGE or eSAGE in their practice. Also, every PCP office will have different patient populations (e.g., socioeconomic status, ethnicities, and co-morbidities) they serve and that may result in different outcomes than we found in our study. In addition, we had three providers who did not complete the provider questionnaire and their answers, if they had completed it, may have altered our results.

In summary, SAGE was easily incorporated into PCP visits, and its use resulted in a significant 6-fold detection of new cognitive conditions/concerns leading to new diagnoses, treatment, or management changes. PCPs identified nearly 4-fold as many patients as having cognitive conditions/concerns if their patient’s SAGE score was in the cognitively impaired range and another 4-fold more patients if their impaired SAGE score was combined with informant reports of significant cognitive declines over a year. PCPs felt SAGE influenced their decision to further evaluate for cognitive impairment, led to more confidence regarding the presence or absence of cognitive impairment, and 86% would recommend its use to colleagues.

## Data availability statement

The raw data supporting the conclusions of this article will be made available by the authors, without undue reservation.

## Ethics statement

The studies involving humans were approved by The Ohio State University’s Biomedical Sciences Institutional Review Board. The studies were conducted in accordance with the local legislation and institutional requirements. The ethics committee/institutional review board waived the requirement of written informed consent for participation from the participants or the participants’ legal guardians/next of kin because only standard of care assessments were used (no experimental tests or assessments). SAGE is used as standard of care for screening for cognitive impairment in the intervention site. This investigational study met institutional requirements for conduct of human subjects research and was pre-registered on ClinicalTrials.gov (identifier: NCT04063371).

## Author contributions

DS: Conceptualization, Formal analysis, Funding acquisition, Methodology, Supervision, Writing – original draft, Writing – review & editing. NV: Conceptualization, Formal analysis, Investigation, Methodology, Project administration, Writing – review & editing. HN: Conceptualization, Formal analysis, Methodology, Writing – review & editing. RW: Data curation, Investigation, Writing – review & editing. AC: Data curation, Investigation, Writing – review & editing. CN: Project administration, Writing – original draft, Writing – review & editing.
