# Supplementary material for: Self-administered gerocognitive examination (SAGE) aids early detection of cognitive impairment at primary care provider visits
Source: Front Med (Lausanne). 2024 Jun 13;11:1353104. doi: 10.3389/fmed.2024.1353104 (PMC11208700; doi:10.3389/fmed.2024.1353104)
Supplement: Supplementary file 1 [file Data_Sheet_1.pdf]

Supplemental Figure 1. Questionnaire for Provider

Provider Questions

Please circle the most applicable answer.

1. The SAGE test was useful.

1	2	3	4	5
Strongly Disagree	Disagree	Neutral	Agree	Strongly Agree

2. The SAGE test was easy to incorporate into an office visit.

1	2	3	4	5
Strongly Disagree	Disagree	Neutral	Agree	Strongly Agree

3. Did the results of the SAGE test influence your decision to further evaluate for cognitive impairment?

Yes No

4. Did the results of the SAGE test lead to more confidence regarding the presence or absence of cognitive impairment?

Yes No

5. SAGE administration did not take up much of the physician's time.

1	2	3	4	5
Strongly Disagree	Disagree	Neutral	Agree	Strongly Agree

6. **SAGE administration did not take up much of the support staff's time.**

1	2	3	4	5
Strongly Disagree	Disagree	Neutral	Agree	Strongly Agree

7. Would you recommend the SAGE test during office visits for other colleagues?

Yes No

Additional Comments:

____

____

____

____
